# Bacteria of the Candidate Phylum TM7 are Prevalent in Acidophilic Nitrifying Sequencing-Batch Reactors

**DOI:** 10.1264/jsme2.ME14052

**Published:** 2014-09-20

**Authors:** Akiko Hanada, Takashi Kurogi, Nguyen Minh Giang, Takeshi Yamada, Yuki Kamimoto, Yoshiaki Kiso, Akira Hiraishi

**Affiliations:** 1 Department of Environmental and Life Sciences, Toyohashi University of Technology, Tempaku-cho, Toyohashi 441–8580, Japan; 2 EcoTopia Science Institute, Nagoya University, Furo-cho, Chikusa-ku, Nagoya, 464–8601, Japan

**Keywords:** acidophilic nitrification, ammonia oxidation, TM7 bacteria, *amoA* gene

## Abstract

Laboratory-scale acidophilic nitrifying sequencing-batch reactors (ANSBRs) were constructed by seeding with sewage-activated sludge and cultivating with ammonium-containing acidic mineral medium (pH 4.0) with or without a trace amount of yeast extract. In every batch cycle, the pH varied between 2.7 and 4.0, and ammonium was completely converted to nitrate. Attempts to detect nitrifying functional genes in the fully acclimated ANSBRs by PCR with previously designed primers mostly gave negative results. 16S rRNA gene-targeted PCR and a subsequent denaturating gradient gel electrophoresis analysis revealed that a marked change occurred in the bacterial community during the overall period of operation, in which members of the candidate phylum TM7 and the class *Gammaproteobacteria* became predominant at the fully acclimated stage. This result was fully supported by a 16S rRNA gene clone library analysis, as the major phylogenetic groups of clones detected (>5% of the total) were TM7 (33%), *Gammaproteobacteria* (37%), *Actinobacteria* (10%), and *Alphaproteobacteria* (8%). Fluorescence *in situ* hybridization with specific probes also demonstrated the prevalence of TM7 bacteria and *Gammaproteobacteria*. These results suggest that previously unknown nitrifying microorganisms may play a major role in ANSBRs; however, the ecophysiological significance of the TM7 bacteria predominating in this process remains unclear.

Nitrification is an important biological process not only in the global nitrogen cycle, but also in agriculture and wastewater treatment technology. The oxidation of ammonia to nitrite is the first and rate-limiting step of nitrification, which is performed by two different phylogenetic groups of microorganisms, *i.e.*, ammonia-oxidizing archaea (AOA) and bacteria (AOB). The subsequence oxidation of nitrite to nitrate is mediated by nitrite-oxidizing bacteria (NOB). AOA members, most of which have been detected as uncultured environmental clones, have been assigned to the phylum *Thaumarchaeota* (29, 31), while the main AOB groups belong to the classes *Betaproteobacteria* and *Gammaproteobacteria*. Although these phylogenetic groups of ammonia oxidizers are widely distributed in nature, AOA and AOB may have different affinities to ammonia as the substrate and ecological niches (31, 57, 63). Archaeal *amoA* genes, coding for the ammonia monooxygenase (AMO) α subunit, are abundant in most soils, suggesting that AOA as well as AOB have important roles in ammonia oxidation in terrestrial environments (49, 63). Studies on the relative abundance of AOA and AOB in wastewater treatment systems have been undertaken in recent years (for a review, see ref. 50), and information on this subject is fragmentary and controversial (42, 60, 67, 76, 80, 82, 83).

Ammonia oxidation in wastewater treatment was previously considered to occur at an approximately neutral pH and was inhibited under acidic conditions. One of the main reasons for this was that the acidification of wastewater, for example, due to the accumulation of nitrite and nitrate as the products of nitrification, reduces the bioavailability of ammonia by ionization (25, 26, 70). Furthermore, free nitric acid has been shown to negatively affect the growth and activity of nitrifying microorganisms at low pHs (3). However, a recent study on the biodegradation of *N,N-*dimethylformamide by a mesh-filtration bioreactor demonstrated that nitrogen removal occurred under strongly acidic conditions (41). The oxidation of ammonia has also been detected in acidic fen (33) and acidic soils (40, 51, 52, 61, 79, 84), in which AOA rather than AOB are responsible for this activity. A chemolithotrophic, obligately acidophilic thaumarchaeal ammonia oxidizer, “*Candidatus* Nitrosotalea devanaterra,” was previously obtained from nitrifying acidic agricultural soil (48). Nevertheless, little or no information is currently available on the nitrifying activity and microorganisms involved in nitrogen removal from acidified wastewater.

In the present study, we successfully constructed acidophilic nitrifying sequencing-batch reactors (ANSBRs) capable of nitrification in artificial mineral wastewater at pH 4.0 and below. The main aims of this study were to estimate the ability of ANSBRs to nitrify and elucidate microbial community dynamics in the ANSBR system. We employed 16S rRNA gene-targeted PCR and denaturating gradient gel electrophoresis (DGGE), 16S rRNA gene cloning and sequencing, fluorescence *in situ* hybridization (FISH), and real-time quantitative PCR (qPCR) targeting 16S rRNA and *amoA* genes to determine microbial community dynamics. We herein demonstrated that members of the candidate phylum TM7, a major bacterial lineage currently known only from environmental sequence data (37, 38), as well as those of the class *Gammaproteobacteria*, were prevalent in ANSBRs at the fully acclimated stage.

## Materials and methods

### Construction and operation of ANSBRs

Two CULSTIR^®^ flasks (Shibata Scientific Technology Ltd., Soka, Japan) having a working volume of 1 L were used to construct ANSBRs. The flasks were seeded with activated sludge taken from the sewage treatment plant of Toyohashi University of Technology to give an initial concentration of mixed liquor suspended solids (MLSS) as 2,000 mg L^−1^. The activated sludge plant from which the sample was taken treats domestic sewage discharged within the university campus and the main aeration tanks are maintained under neutral conditions. The mixed liquor suspension used as the seed had a pH of 7.1 when used. One of the reactors, designated ANSBR 1, was loaded with synthetic mineral wastewater SMW1, which contained (per L) 107 mg NH_4_Cl, 168 mg NaHCO_3_, 136 mg KH_2_PO_4_, 30 mg MgSO_4_·7H_2_O, 10 mg CaCl_2_·2H_2_O, and 1 mL of trace element solution SL8 (8). The other reactor, designated ANSBR 2, was loaded with SMW1 medium supplemented with 0.01% Bacto^®^ yeast extract (Beckton and Dickinson and Company [BD], Franklin Lakes, NJ, USA). Both of these media were adjusted to pH 4.0 and autoclaved before use. Furthermore, 0.5 mM cycloheximide was added to the media to suppress the growth of eukaryotic microorganisms. The reactors were operated at 25°C for 98 d with a batch cycle of 3–4 d, and half of the supernatant of the reactors was exchanged with fresh medium in each batch cycle. The mixed liquor in ANSBRs was always rotated on a magnetic stirrer at 160 rpm and aerated with an air pump to give a dissolved oxygen (DO) tension at 3–5 mg L^−1^. Experiments to construct ANSBRs 1 and 2 were performed in three independent runs.

### Analysis of physicochemical parameters

The pH of the reactors was measured with a Horiba pH meter. Dissolved oxygen tension was measured with a DO meter. MLSS was determined by measuring the optical density at 660 nm (OD_660_) and using a linear regression equation showing the relationship between OD_660_ and dry weight of sludge as measured by a standard method (4).

### Measurement of nitrifying activity

The nitrifying activity of ANSBRs was determined by monitoring the consumption of ammonium as the substrate and production of nitrate as the end product in each batch cycle. Mixed liquor samples were taken from ANSBRs and centrifuged to save the supernatant. The supernatant samples were filtered through membrane filters (pore size, 0.2 μm) and directly subjected to analyses. Vial tests with mixed liquor samples taken from ANSBR 1 on d 84–98 were performed to determine nitrifying activities. These samples were introduced into 60-mL vials containing 10 mL of SWM1 medium adjusted to pH 3.5 to 7.0, and then incubated aerobically with vigorous shaking at 25°C for 24–72 h, followed by the measurement of ammonium and nitrate. In some cases, vial tests with 10 mM chlorate, a potent inhibitor of nitrite oxidation (7), were performed to examine the production of nitrite from ammonium. The concentration of ammonia/ammonium ions was measured using indophenol spectrophotometry (4). Nitrite and nitrate were measured by ion chromatography as described previously (71). The apparent nitrification rate (ANR) was determined based on the maximum velocity of the conversion of ammonium to nitrate in a batch cycle, *i.e.*, the average ammonia consumption and nitrate production rates.

### Direct cell counting

Regarding cell counting, 5 mL of a mixed-liquor sludge sample from ANSBRs was added to a BD Falcon™ tube, sonicated for 100 s (20 kHz; output power 50 W), and diluted with filter-sterilized phosphate-buffered saline (pH 7.0). Aliquots (10–50 μL) of these diluted samples were taken and used for direct cell counting. The direct total count was measured by epifluorescence microscopy with 4,6-diamidino-2-phenylindole (DAPI) or SYBR Green I (Invitrogen Corporation, Carlsbad, CA, USA) staining as described (22, 76). A direct viable count was also obtained using a Molecular Probe LIVE/DEAD^®^ *Bac*Light™ Viability kit (Invitrogen) as described (24, 81). Stained specimens were observed under an Olympus model BX-50 phase-contrast/epifluorescence microscope equipped with an Olympus DP70 digital camera (Olympus Corporation, Tokyo, Japan).

### Fluorescence *in situ* hybridization (FISH)

The biomass from ANSBRs was taken into BD Falcon™ tubes, prepared as noted above for total cell counting, and subjected to FISH analyses. Six oligonucleotide probes previously designed for the specific detection of microorganisms at the domain, phylum, and class levels were commercially synthesized (Life Technologies Corporation, Carlsbad, CA, USA) and used together for multicolor identification. An equimolar mixture of the three bacterial probes, EUB338 I, EUB338 II, and EUB338 III (2, 14), which targeted members of the domain *Bacteria*, were 5′-labeled with Alexa Fluor^®^ 488. Three probes, ARC915, TM7905, and GAM42a, which targeted the domain *Archaea* (68), the candidate phylum TM7 (38), and class *Gammaproteobacteria* (54), respectively, were 5′-labeled with the cyanine dye Cy3 or Alexa Fluor^®^ 546. Hybridization was performed under optimized conditions using standard FISH protocols as described (38, 54, 68). The FISH-stained biomass was counterstained with DAPI and observed under the Olympus epifluorescence microscope system as described above. FISH images were taken and analyzed using the ImageJ version 1.47 program (http://rsb.info.nih.gov/ij/).

### DNA extraction and purification

The bulk DNA of the biomass collected from ANSBRs was extracted as previously described (35, 43). Extracted DNA was further purified by a standard procedure including phenol/chloroform/isoamyl alcohol (25:24:1, v/v/v) and RNase A treatment and ethanol precipitation (56).

### Standard PCR assays for 16S rRNA and nitrifying functional genes

PCR experiments for the amplification of 16S rRNA and *amoA* genes from ANSBRs were performed with previously reported pair primer sets. The primer sets used were 27f/1492r (46) for bacterial 16S rRNA genes, A21f/1492r, A21f/A958r (16), and A109f/A934b (29) for archaeal 16S rRNA genes, amoA-1F/amoA-2R (65) for betaproteobacterial *amoA* genes, amoA-3F/amoB-4R for gammaproteobacterial (*Nitrosococcus*) *amoA* genes (64), and Arch-amoAF/Arch-amoAR (22), amo111F/amo643R (75), and CrenamoA23f/CrenamoA616r (74) for archaeal *amoA* genes. In addition, the presence of the nitrite oxidation genes *nxrA* from *Nitrobacter* and *nxrB* from *Nitrospira* was determined using F1norA/R1norA (62) and nxrBF916/nxrBR1237 (53) primers, respectively. Amplification was performed using an AmpliTaq Gold *Taq* DNA polymerase kit (Applied Biosystems, Foster City, CA, USA) and Takara Thermal Cycler (Takara Bio, Otsu, Japan). The PCR profile consisted of activation of the polymerase at 94°C for 10 min and 30 cycles of denaturation at 94°C for 1 min, annealing at 53°C for 1 min, and extension at 72°C for 1 min, followed by a 5-min extension at 72°C. The annealing temperature was also changed between 45 and 60°C for the amplification of *amoA*. PCR products were detected by 1.5% agarose gel electrophoresis with ethidium bromide staining.

### Real-time qPCR

To determine the copy numbers of bacterial 16S rRNA and *amoA* genes in ANSBRs, real-time qPCR assays were performed using a primer set of 341f/938r (46) and amoA-1F/amoA-2R (65), respectively, and Light Cycler (Roche, Basal, Switzerland) with SYBR Premix Ex *Taq* Perfect Real Time (Takara) as described (78). The reaction mixture contained 1 or 10 ng of template DNA according to the manufacturer’s instructions. The standards to quantify the 16S rRNA and *amoA* gene copies were prepared using PCR products from *Escherichia coli* IAM 12119^T^ and *Nitrosomonas europaea* IFO 14298^T^ (NBRC 14298), respectively. The PCR procedure consisted of an initial 5-s denaturation step at 95°C followed by 40 cycles of 5 s denaturation at 95°C, 10 s annealing at 50°C for 16S rRNA genes and 55°C for AOB *amoA*, and 45 s extension at 72°C. All PCR amplifications were performed in triplicate. Melting curve analysis was performed to confirm the specificity of the results of real-time qPCR. Amplicons were also detected by agarose gel electrophoresis with ethidium bromide staining.

### PCR-DGGE

Bulk DNA samples extracted from ANSBRs on d 0, 14, 21, 35, 49, 63, and 91 were used for PCR-DGGE. The variable region V3 of bacterial 16S rRNA genes, corresponding to positions 341–534 in *E. coli* 16S rRNA (10), was PCR-amplified using the forward primer GC341f with a GC-clamp on the 5′ terminus and the reverse primer 534r as described previously (59). Amplification was performed using an AmpliTaq Gold *Taq* DNA polymerase kit and Takara Thermal Cycler. The PCR profile consisted of 10 min activation of the polymerase at 94°C and 40 cycles of 1 min denaturation at 94°C, 1 min annealing at 53°C, and 1 min extension at 72°C, followed by 5 min extension at 72°C. Amplicons were checked by agarose gel electrophoresis with ethidium bromide staining, purified with a MicroSpin S-HR400 Column (Amersham Biosciences, Piscataway, NJ, USA), and analyzed by DGGE using a Bio-Rad DCode™ system (Bio-Rad Laboratories, Hercules, CA, USA) as described previously (24). DGGE bands were detected by staining with ethidium bromide, photographed, and analyzed for their intensity using the ImageJ version 1.47 program. DNA fragments from the major DGGE bands were extracted and purified for sequencing as described (24).

### Construction of a 16S rRNA gene clone library

DNA samples from ANSBR 1 on d 91 were used to construct a 16S rRNA gene clone library. 16S rRNA gene fragments from the purified DNA were PCR-amplified using a PCR primer set of 27f and 1492r as described above. The PCR products were purified using the Qbiogene Geneclean Spin kit (MP Biomedicals, Santa Ana, CA, USA) and subcloned using the pTBlue Perfectly Blunt cloning kit (Novagen, Madison, WI, USA) and *E. coli* JM109 competent cells (Takara). Plasmid DNA was purified using a plasmid extraction kit (Sigma-Aldrich, St. Louis, MO, USA) according to the manufacturer’s instructions and subjected to the analyses described below.

### Phylogenetic analyses of 16S rRNA gene clones

16S rRNA gene clones as plasmid inserts were re-amplified with the PCR primers 27f/1492, digested with the restriction enzymes *Hae*III, *Hha*I, or *Msp*I, and separated by MetaPhor™ agarose gel electrophoresis to analyze restriction fragment length polymorphisms (RFLP), as described previously (34). Clones showing different RFLP patterns were classified into different operational taxonomic units (OTUs). 16S rRNA gene clones were sequenced using a BigDye Terminator v3.1 cycle sequencing kit (Applied Biosystems) and an Applied Biosystems 3130xl genetic analyzer according to the manufacturer’s instructions. Sequence data were compiled with the GENETYX-MAC ver. 17 program (GENETYX, Tokyo, Japan) and compared to those available from the public database using the BLAST search system (1) and RDP-11 Seqmatch algorithm with the option of the type-strain match (13). Chimeric sequences were examined by a partial treeing analysis (39). The multiple alignments of sequences were performed with the CLUSTAL X version 2.0 program (47), and neighbor-joining (NJ) phylogenetic trees (66) based on Kimura’s two-parameter model (44) were re-constructed using MEGA software version 5.0 (72). The tree topology was evaluated by bootstrap resampling with 1,000 replicates (20).

### Nucleotide sequence accession numbers

The 16S rRNA gene sequences determined in this study have been deposited under the DDBJ accession numbers AB809939 to AB809970.

## Results

### Characteristics and performance of ANSBRs

**Fig. 1:**
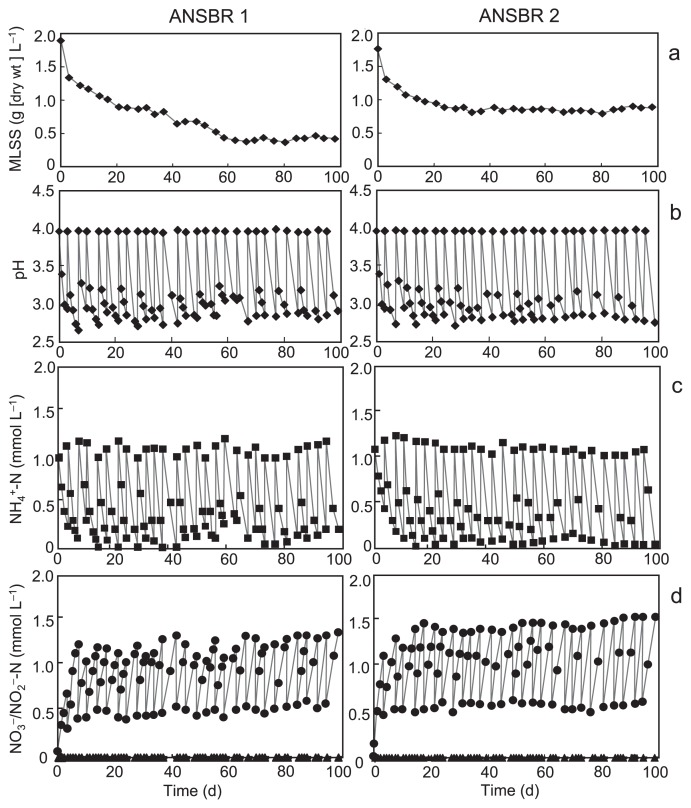
Physicochemical characteristics and nitrification performance of ANSBRs 1 (left) and 2 (right) during 98 d of operation. (a), MLSS concentration; (b), pH; (c), NH_4_^+^-N concentration; (d), NO_3_^−^-N (circles), and NO_2_^−^-N (triangles) concentrations. Fig. 1b–1d show changes in the parameters in every batch cycle (at least two data points at the start [just after the addition of the substrate] and at the end of a batch cycle).

We constructed ANSBRs 1 and 2 in three respective independent experiments, and found that the performance of the two types of reactors regarding nitrification was good, with similar features being observed in the three runs. The typical physicochemical features of ANSBRs 1 and 2 during the overall period of operation are shown in Fig. 1. In ANSBR 1, MLSS lowered gradually with time and steadied at 25% of the initial concentration after 2 months of operation, whereas ANSBR 2 kept 50% of the initial concentration of the biomass by the end of operation because of the possibly stimulating effects of the added yeast extract on chemoorganotrophic growth (Fig. 1a). In both reactors, the pH varied between 2.7 and 4.0 (Fig. 1b), and the net amount of ammonium added was almost completely consumed and converted to nitrate by the end of each batch cycle under these acidic conditions (Fig. 1c). Nitric acid was not produced in detectable amounts at any stage. Nitrification decayed when the concentration of ammonium fed to the reactors in each batch cycle was elevated to 2 mM (data not shown).

**Fig. 2:**
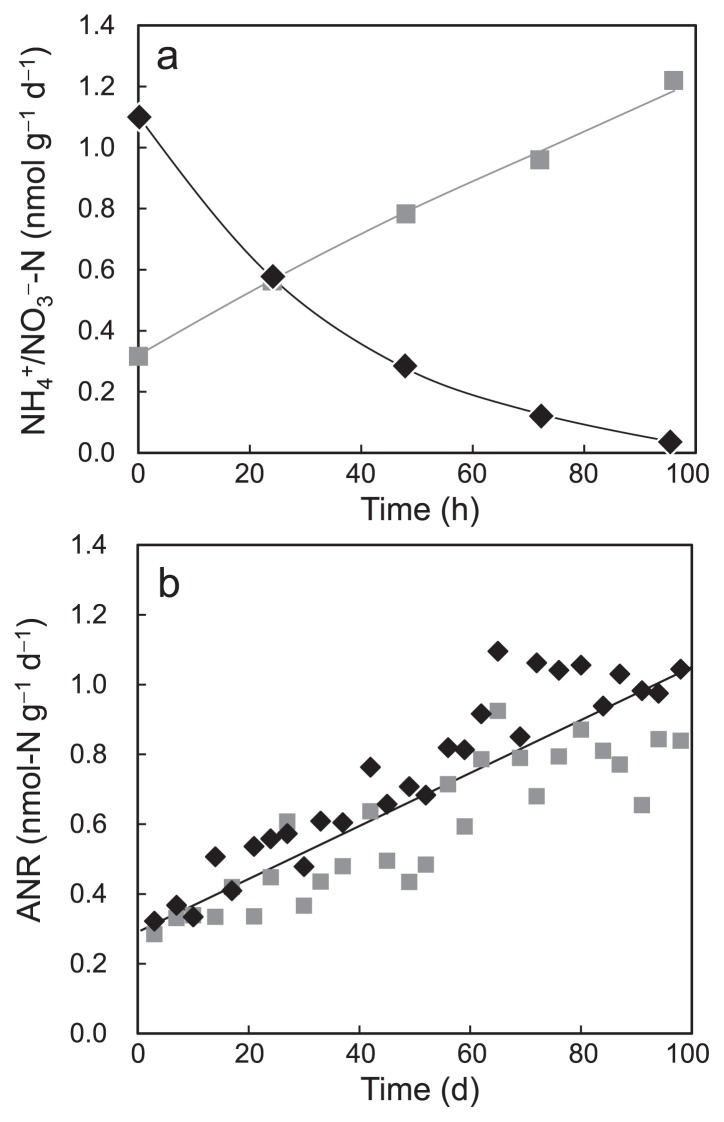
Typical profiles of ammonium removal and nitrate production in a batch cycle of ANSBR 1 at the fully acclimated stage (a) and the relationship between ANR and the operational time (b). Symbols in (a): closed circles, NH_4_^+^-N concentration; squares, NO_3_^−^-N concentration. Symbols in (b): diamonds, ANR based on ammonium removal; squares, ANR based on nitrate production. The regression equation based on all plotted data in Fig. 2b is given by: *y* = 0.0071*x* + 0.307 (*R*^2^ = 0.8722).

A typical batch profile of fully acclimated ANSBR 1 showing a reverse relationship between ammonium removal and nitrate production is shown in Fig. 2a. The ANRs in every batch cycle were calculated on the basis of this relationship. The recorded ANRs increased gradually with the operational time and reached approximately 1.0 mmol-N g-MLSS^−1^ d^−1^ at the end of operation (Fig. 2b). Although this ANR value was lower than those previously reported for nitrogen removal in the standard activated sludge process (19, 58), the fully acclimated ANSBR process showed similar ANRs in the three different runs, indicating the reproducibility of the nitrification process adapted to acidic conditions.

In ANSBR 1, the cumulative amount of NO_3_^−^-N produced corresponded to 77% of NH_4_^+^-N added to the ANSBR (data not shown). To determine whether the nitric acid produced from ammonium was lost via evaporation under acidic conditions, we attempted to detect nitric acid by inhibiting nitrite oxidation with 10 mM chlorate. However, this attempt was unsuccessful because the consumption of ammonium itself was inhibited by the addition of chlorate at pH 4.0. Although the fate of the remaining 23% of NH_4_^+^-N remains unknown, it may be explained by assuming that denitrification occurred in ANSBR 1. Autotrophic denitrification with the formation of NO and N_2_O as byproducts by neutrophilic ammonia oxidizers has been well documented (for reviews, see refs. 5 and 11). On the other hand, ANSBR 2 produced a cumulative amount of NO_3_^−^-N corresponding to 105% of NH_4_^+^-N added, possibly because some nitrogen compounds derived from the added yeast extract were additionally converted to nitrate. Since the total nitrogen content of Bacto^®^ yeast extract is approximately 11% on a dry wt basis (6), ANSBR 2 may have been supplied with 5.5 mg-N from this component in each batch cycle. Therefore, ANRs as determined based on the averages of the net amounts of ammonium added and nitrate produced may have been underestimated.

**Fig. 3:**
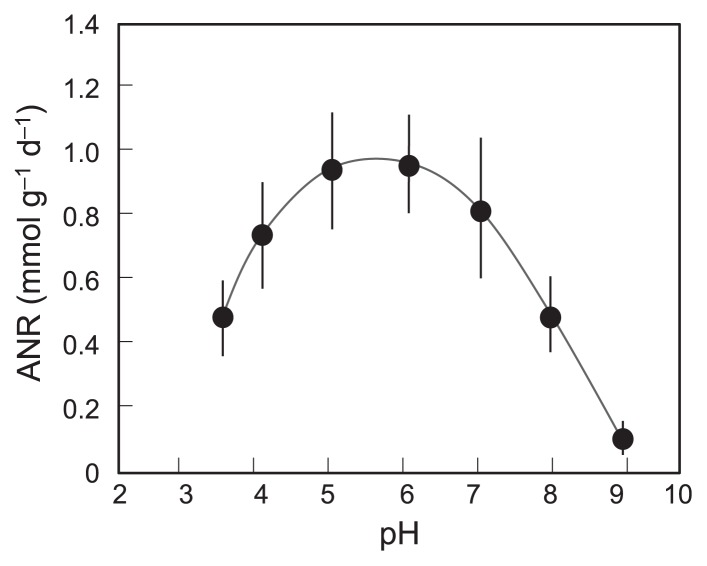
Apparent nitrification rate of ANSBR 1 by vial testing with SWM1 medium as a function of external pH. The pH of the medium was adjusted to 3.5–4.0 by adding HCl. The medium having other pHs was prepared with 50 mM MES for pH 5–6, 50 mM MOPS pH 7, and 50 mM Tricine for pH 8–9. The data show the averages (± standard deviations) for five different determinations for ANSBR 1 on d 84–98.

To establish whether the nitrification process we constructed was actually acidophilic, the effects of pH on nitrifying activity were studied in vials using the biomass taken from ANSBR 1 on d 84–98. As shown in Fig. 3, the maximum ANR was recorded at pH 5–6, thereby confirming that the nitrifying community acclimated was acidophilic, but not acid-tolerant.

### Appearance of biomass and direct total counts

The sewage sludge used as the seed for constructing ANSBRs was brownish gray; however, acclimation of the reactors turned the sludge biomass beige to bright gray. Compact sludge flocs were observed under a phase-contrast microscope (Fig. S1a), and microorganisms at the fully acclimated stage were embedded in extracellular matrices that were weakly stained with SYBR Green (Fig. S1b). Although these flocs were not easily broken by sonication, possibly because of the presence of extracellular substances, we temporarily used a sonic treatment for 100 s to disperse microbial cells for direct cell counting.

The direct total counts in ANSBRs as measured by DAPI or SYBR Green staining varied in proportion to the concentration of MLSS, ranging from 0.8 to 3.1×10^9^ mL^−1^ in ANSBR 1 and from 1.3 to 3.1×10^9^ mL^−1^ in ANSBR 2. In the *Bac*Light kit-using assays, the biomass that stained fluorescent red with propidium iodide accounted for 14 to 24% of the total biomass in ANSBRs during the overall period of operation. These results indicated that, although the concentration of the biomass in ANSBRs decreased with the operation time, 80% of the ANSBR population on average was constantly viable.

### PCR detection of 16S rRNA and *amoA* genes

We performed PCR assays for 16S rRNA and *amoA* genes from ANSBRs 1 and 2 for each week of operation. Both bacterial and archaeal 16S rRNA genes could be detected in ANSBRs at all stages of operation, although the amplification of archaeal 16S rRNA genes was possible only with the primer set of A109f/915r (data not shown). The PCR signals of archaeal 16S rRNA fragments were less than 3% of those of bacterial 16S rRNA genes during the overall period of operation, thereby suggesting that *Archaea* constituted a minor population in ANSBRs.

Bacterial *amoA* gene fragments with amoA-1F/amoA-2R could be PCR-amplified in both ANSBRs with a decrease being observed in the intensity of PCR signals with the operation time (Fig. S2a). No AOB *amoA* genes with a primer set of amoA-1F/amoA-2R or amoA-3F/amoB-4R were detected at the end of operation (data not shown). Real-time qPCR assays for ANSBR 1 showed that, whereas the number of bacterial 16S rRNA gene copies was relatively constant, the number of AOB *amoA* gene copies with primers amoA-1F/amoA-2R rapidly reduced with time (Fig. S2b). This result suggested that the role of AOB, as detected by the PCR primer set used, became less significant with the operation time. Attempts to detect archaeal *amoA* genes in ANSBRs with Arch-amoAF/Arch-amoAR or amo111F/amo643R mostly gave negative results during the overall period of operation. We detected weak PCR signals with the primer set of CrenamoA23f/CrenamoA616r in ANSBR 1 at some stages of operation (data not shown); however, the amount of these amplicons was too low to accomplish subcloning and sequencing. Standard PCR assays with any primer set failed to detect AOA *amoA* at the end of operation, similar to AOB *amoA*. Fully acclimated ANSBRs gave no PCR products of the nitrite oxidation gene *nxrA* of *Nitrobacter*, while only faint PCR signals of *Nitrospira nxrB* were detected.

### PCR-DGGE profiles

**Fig. 4:**
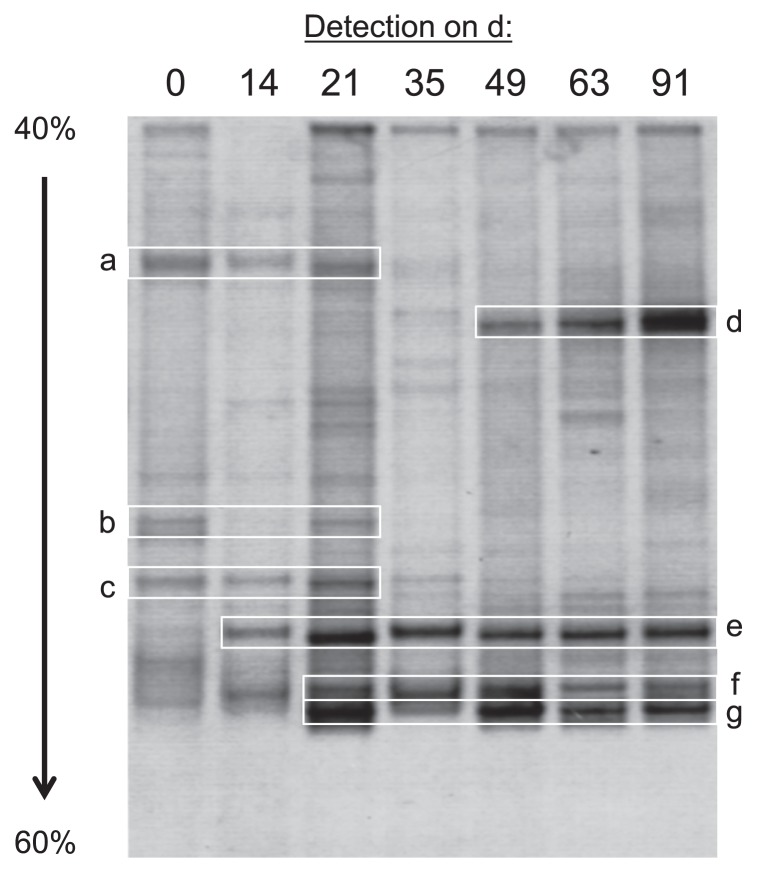
Changes in 16S rRNA gene (V3 region)-targeted PCR-DGGE profiles of ANSBR 1 with the operational time. Major DGGE bands (designated a–g) were cut off from the gel, sequenced, and phylogenetically analyzed. Detailed information on the phylogenetic assignment of the DGGE clones is shown in Table S2. Similar PCR-DGGE patterns were obtained from ANSBR 2 (data not shown).

Based on the results of PCR experiments described above, we performed PCR-DGGE analyses targeting the 16S rRNA genes (V3 region) of bacteria as the major populations to roughly estimate bacterial community dynamics in ANSBRs. The DGGE patterns from ANSBR 1 markedly changed with the operational time, and four major bands became conspicuous at the fully acclimated stage (Fig. 4). These DGGE clones were assigned with members of TM7 (bands grouped as d), *Gammaproteobacteria* (those grouped as e), *Actinobacteria* (those grouped as f), and *Alphaproteobacteria* (those grouped as g) (Table S1). An image analysis of the d 91 lane on the gel showed that the intensity ratio of bands d, e, f, and g was 38:26:18:18. Similar results for PCR-DGGE profiling were obtained with ANSBR 2 (data not shown). These results demonstrated the prevalence of TM7 bacteria in the ANSBR system at the fully acclimated stage.

### Clone library analysis

To confirm the results of PCR-DGGE profiling, we constructed a 16S rRNA gene clone library from ANSBR 1 on d 91. More than 300 clones were obtained from this library, sequenced, and examined for chimeric artifacts. Positive 212 clones as complete sequences thus obtained were grouped into 35 OTUs (designated OTU 1 to OTU 35) on the basis of combined data on *Hae*III-, *Hha*I-, and *Msp*I-digested RFLP patterns, and 1 to 22 clones of each OTU were sequenced. The clones within a single OTU had almost the same sequence at 99.8–100% levels of similarity, and those of different OTUs were different from one another at < 99.0% similarity levels. An exception was that OTU 33 and OTU 34 were very similar to each other at a 99.7% level of similarity.

Phylogenetic analyses using BLAST and RDP Seqmatch revealed that the clones of the 35 OTUs could be assigned to 9 phyla, *i.e.*, *Acidobacteria*, *Actinobacteria*, *Armatimonadetes*, *Cyanobacteria*, *Firmicutes*, *Nitrospira*, *Planctomycetes*, *Proteobacteria* (*Alpha-*, *Beta-*, and *Gammaproteobacteria*), and TM7 (Table 1). Most of the clones belonged to *Gammaproteobacteria* (37.3%) and TM7 (33.0%). Significant proportions of all clones were represented by *Actinobacteria* (10.4%) and *Alphaproteobacteria* (8.0%). These results were consistent with those of PCR-DGGE profiling.

**Fig. 5:**
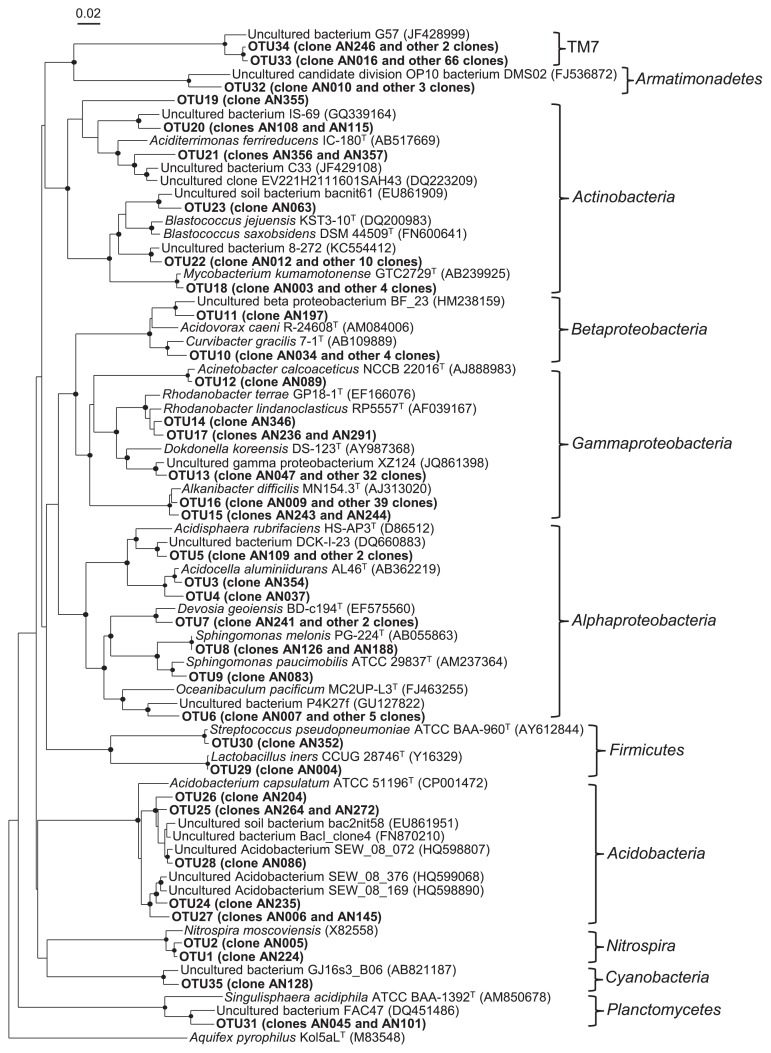
Neighbor-joining distance matrix tree showing phylogenetic positions of the 16S rRNA gene clones obtained from ANSBR 1 on d 91. Representative clones of the 35 OTUs are shown with their closest relatives of established species and/or uncultured bacteria whose sequences were retrieved from the public database. The sequence of *Aquifex pyrophilus* (M83548) was used as the outgroup to root the tree. The nodes supported by a bootstrap value of more than 80% are shown by closed circles. Bar = 2% nucleotide substitution rate (*K*_nuc_).

An NJ phylogenetic tree re-constructed based on 16S rRNA gene clones and their closest relatives retrieved from the database is shown in Fig. 5. Out of the 207 clones incorporated, 65% (including all of the TM7 phylum and *Acidobacteria*, 77% of *Actinobacteria*, and 42% of the *Gammaproteobacteria*) exhibited less than 95% similarity to their closest relatives as the established species. Therefore, it was difficult to infer from the phylogenetic tree what the physiological nature of the uncultured bacteria was as the source of these major clones, except that those of *Acidobacteria* were most likely acidophilic. Nevertheless, several other clones clustered with the genera consisting of acidophilic species, *i.e.*, *Acidocella*, *Acidisphaera*, *and Aciditerrimonas*, and 53% of the gammaproteobacterial clones proved to be close at > 97% similarity to *Alkanibacter difficilis* (AJ313020), for which the culture medium was optimized at pH 5.0 (23). Furthermore, few clones were tightly clustered with the nitrite-oxidizer *Nitrospira moscoviensis* (X82558). No clones clustered with the previously known species of AOB and alphaproteobacterial NOB at >90% levels.

Dinis *et al.* (18) reported that the TM7 bacteria so far described can be classified into subdivisions 1 and 2 within the phylum TM7. In this context, we constructed another NJ tree based on the 16S rRNA gene sequences of the TM7 clones detected in this study and of uncultured TM7 bacteria retrieved from the database, and found that our TM7 clones were positioned in subdivision 1 (Fig. S3).

### rRNA-targeted FISH

**Fig. 6:**
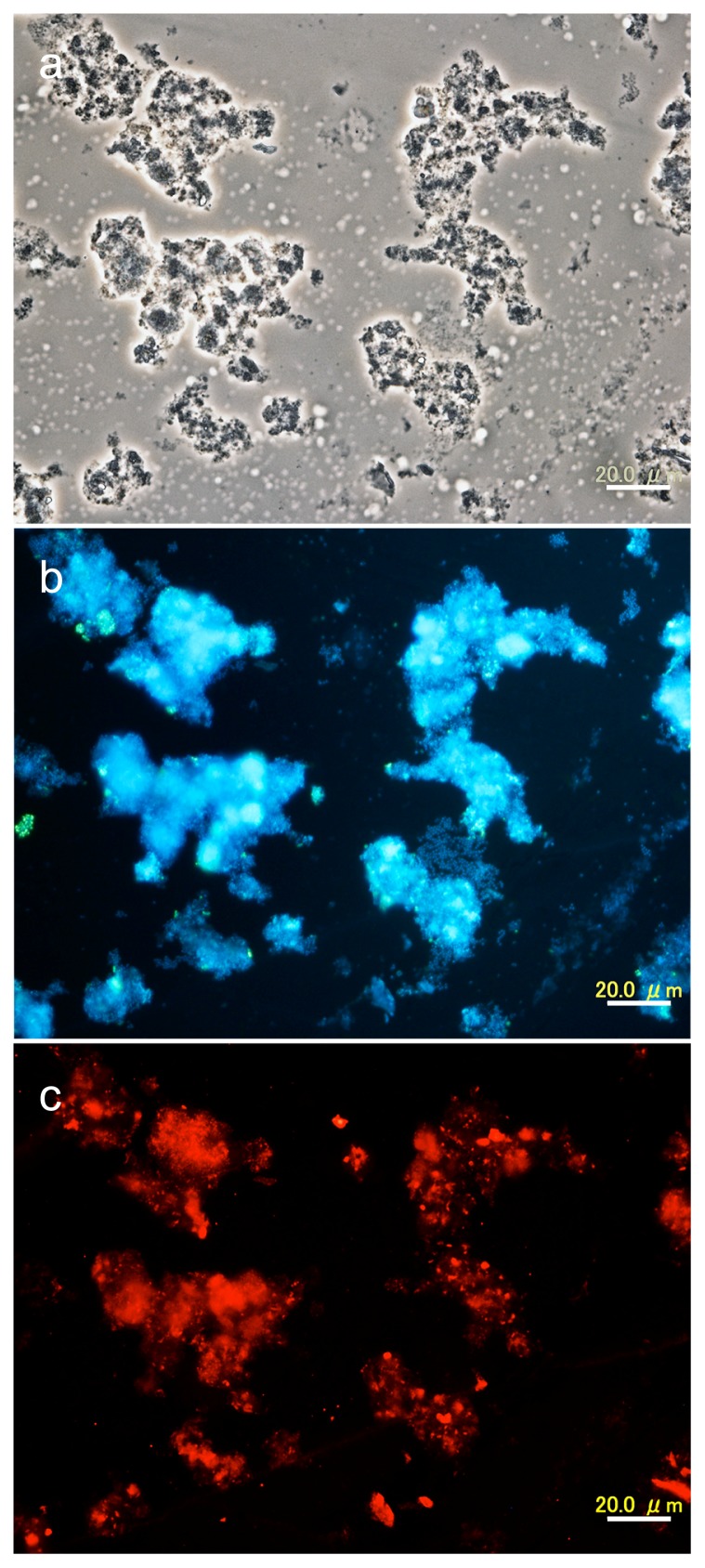
FISH probing of the microbial community of ANSBR 1 in the fully acclimated stage. (a), phase-contrast micrograph; (b), DAPI-stained fluorescence micrograph of the same field as (a); (c) fluorescence micrograph of the same field as (a) showing the biomass stained with the TM7-targeted FISH probe TM9705 (red). Scale bars = 20 μm. Hybridization with the probe TM7905 (labelled with Alexa Fluor^®^ 546) was performed at 46°C for 2 h in the presence of 20% formamide.

To estimate the abundance of different phylogenetic groups of microorganisms in the nitrification process, 16S and 23S rRNA-targeted FISH assays with specific oligonucleotide probes was also performed for ANSBR 1 on d 70–91. FISH probing with a mixture of the EUB338 series and ARCH915 resulted in the detection of 78±5% and 1.0±0.5% of the DAPI- or SYBR-Green-stained total population, respectively (data not shown). This result suggested that members of the domain *Bacteria* constituted the main population of microorganisms in ANSBRs, which was consistent with the results of PCR experiments. FISH probing with TM7905 and GAM42a revealed that the populations of TM7 (Fig. 6b) and *Gammaproteobacteria* (data not shown) accounted for 34±4% and 22±5% of the DAPI-stained populations, respectively. Phase-contrast microscopy (Fig. 6a) and FISH probing (Fig. 6b) showed that the TM7 bacteria as a morphotype of rods to coccobacilli occurred in compact cell aggregates.

## Discussion

Nitrification in wastewater has been described as acid sensitive and generally inhibited at pH 6 and below. Despite these limitations of nitrification, several studies previously reported the occurrence of nitrification in acidic soils (30, 40, 51, 52, 61, 69, 79, 84), an acidic fen (33), and acidified wastewater environments (27, 28, 41). In the present study, we successfully constructed ANSBRs capable of the complete conversion of ammonium to nitrate at pH 4 and below. Although the average ANR at the acclimated stage (*ca.* 1.0 mmol-N g-MLSS^−1^ d^−1^) was lower than those found in the standard nitrifying process (19, 58), the maximum activity of our ANSBR system was observed at pH 5–6. Therefore, we concluded that acidophilic, but not acid-tolerant nitrifying communities were constructed in our system.

The bioavailability of ammonia as the substrate for AMO is reduced by ionization under acidic conditions (25, 26, 70), and high concentrations of free nitric acid also negatively affect the growth and activity of nitrifying microorganisms (3). Therefore, one of the major questions regarding acidophilic nitrification in wastewater is how nitrifiers survive and take up ammonia as the substrate for AMO at low pHs. Although previous studies postulated the existence of neutral or less-acidic microenvironments to explain nitrifying activity under acidic conditions (15, 27), there has so far been no direct evidence to demonstrate this hypothesis. On the other hand, physiological adaptations to low pH have been proposed to explain the occurrence of ammonia oxidation under acidic conditions. Namely, ammonia oxidizers coping with acidic environments may have high affinity to ammonia and express additional functions, *e.g.*, ammonium transporters, that allow them to exhibit nitrifying activity in acidified wastewater (27). An obligately acidophilic thaumarchaeal ammonia oxidizer has been discovered (48), indicating its physiological adaptations to acidic environments. In view of this finding, together with the isolation of an acidophilic nitrite-oxidizing bacterium (32), the complete conversion of ammonium to nitrate may occur even in acidic water, as observed in our ANSBR system. The “omics” approaches to research on these obligately acidophilic nitrifiers should help us to understand the mechanism underlying chemolithotrophic nitrification under acidic conditions.

The culture-independent molecular approaches used in this study revealed that the ANSBR system had an unusual bacterial community structure. One of the most important results of the present study is that members of the candidate phylum TM7, as well as of the class *Gammaproteobacteria*, were prevalent in the fully acclimated ANSBRs, as shown by 16S rRNA gene-targeted PCR-DGGE and clone library analyses. These results were completely supported by FISH probing of phylum- and class-specific rRNA molecules. TM7 bacteria as the uncultured clones have not only been detected in a wide range of natural habitats (37), but have also been commonly found in the human oral microbiome (9, 17, 18, 55). Activated sludge processes and other wastewater treatment systems also harbor TM7 bacteria (12, 18, 38, 73, 77). However, no habitats in which TM7 bacteria constitute the major population of the whole community have so far been reported. For example, the relative abundance of TM7 bacteria to total bacteria in activated sludge as measured by qPCR was *ca.* 3% on average (18). To the best of our knowledge, this study is the first to describe an ecosystem in which TM7 bacteria predominate. Within the candidate phylum TM7, most of the clones retrieved from soil, water, and wastewater environments have been classified into subdivision 1, while those from oral and rumen microbiomes have been classified into subdivision 2 (18). In accordance with this classification system, the TM7 clones detected in this study (OTU 33 and OTU 34) were categorized into subdivision 1. There has been no definite information on the biological significance of TM7 bacteria in the environment, and, because of limited data at this time, our study cannot definitively answer why TM7 bacteria as well as *Gammaproteobacteria* were abundant in the ANSBR system. Furthermore, the detection of a large proportion of actinobacterial clones (*ca.* 10%) in ANSBR was more than expected. Since some members of *Actinobacteria* are capable of heterotrophic nitrification (36), further studies are warranted on this subject.

In the present study, PCR assays targeting the *amoA* genes of both AOA and AOB in the fully acclimated ANSBRs mostly gave negative results. These results suggest that nitrifiers not detectable with conventional PCR primer sets for AOA and AOB may predominant and be involved in the ANSBR system. Our concurrent study showed that nitrification occurred in ANSBRs, even in the presence of streptomycin (45). Interestingly, an atypical nucleotide substitution in 16S rRNA that may be responsible for resistance to streptomycin at the ribosome level is found in most TM7 sequences, similar to those of *Archaea* (38). We confirmed that this unique nucleotide substitution was present in the 16S rRNAs of OTU 33 and OTU 34 as the TM7 phylotypes. Since the PCR experiments revealed the overwhelming majority of bacteria rather than archaea in ANSBRs, one of the possible candidates responsible for ammonia oxidation in the ANSBR system is unusual streptomycin-resistant bacteria, such as TM7 bacteria. In this context, the 16S rRNA gene-targeted high throughput sequencing of streptomycin-resistant ANSBR communities is in progress.

Despite isolation efforts by several laboratories, no axenic cultures of the TM7 phylum have so far been obtained, although microcultivation of soil bacteria under conditions mimicking *in situ* environments resulted in the successful growth of a TM7 bacterium as microcolonies (21). The isolation of TM7 bacteria as axenic cultures and/or metagenomic approaches to the ANSBR microbial community should provide a clearer insight into the biological significance of TM7 bacteria in the acidophilic nitrification process. This is also true for the uncultured *Gammaproteobacteria* that were detected as the major clones in the ANSBR system.

## Supplementary Information



## Figures and Tables

**Table 1:** Phylogenetic assignment of the 16S rRNA gene clones obtained from ANSBR 1 on d 91

Phylum/class	No. of OTUs detected	No. of clones detected	% clones
*Acidobacteria*	5	7	3.3
*Actinobacteria*	6	22	10.4
*Armatimonadetes*	1	4	1.9
*Cyanobacteria*	1	1	0.5
*Firmicutes*	2	2	0.9
*Nitrospira*	2	2	0.9
*Planctomycetes*	1	2	0.9
*Proteobacteria*			
*Alphaproteobacteria*	7	17	8.0
*Betaproteobacteria*	2	6	2.8
*Gammaproteobacteria*	6	79	37.3
TM7	2	70	33.0
Total	35	212	100
