# Supplementary material for: Bacteria of the Candidate Phylum TM7 are Prevalent in Acidophilic Nitrifying Sequencing-Batch Reactors
Source: Microbes Environ. 2014 Sep 20;29(4):353–62. doi: 10.1264/jsme2.ME14052 (PMC4262358; doi:10.1264/jsme2.ME14052)
Supplement: Supplementary file 1 [file 29_353_s1.pdf]

Supplemental figures

Fig. S1. Micrographs of sludge biomass in ANSBR 1 at the fully acclimated sludge. (a), phase-contrast microscopic image; (b), SYBR Green-stained microscopic image of the same filed as (a), showing prominent fluorescent signals of microbial cells embedded in weakly stained extracellular matrices (shown by arrowheads).

Fig. S2. Standard PCR detection of bacterial *amoA* (a) and changes in the copy number of bacterial *amoA* and 16S rRNA genes as measured by real-time qPCR (b) in ANSBR 1 during 63 days of operation. The *amoA* and 16S rRNA genes were PCR-amplified with primer sets of amoA-1F/amoA-2R and 341f/934r, respectively. Symbols in Fig. S1b: closed squares, 16S rRNA gene; open circles, bacterial *amoA*; the plotted data show the averages of triplicate determinations with error bars.

Fig. S3. Neighbor-joining distance matrix tree showing phylogenetic positions of TM7 clones AN016 (OTU 33) and AN246 (OTU 34) within the candidate phylum TM7. The 16S rRNA gene sequences of representatives TM7 bacterium clones were retrieved from public database and incorporated into the tree. Subdivisions 1 and 2 are based on the classification system of Dinis et al. (18), which consist mainly of clones from

natural/wastewater environments and human/animals, respectively. The sequence of *Escherichia coli* (10) is used as an outgroup to root the tree. The nodes supported by a bootstrap value of more than 80% are shown by closed circles. Bar = 2% nucleotide substitution rate (K_{nuc}).

Fig. S1

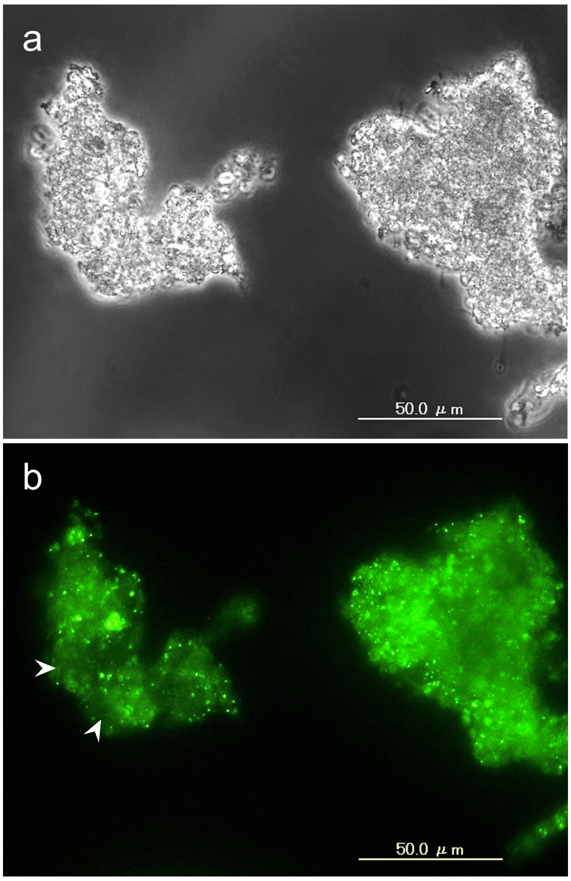

Fig. S2

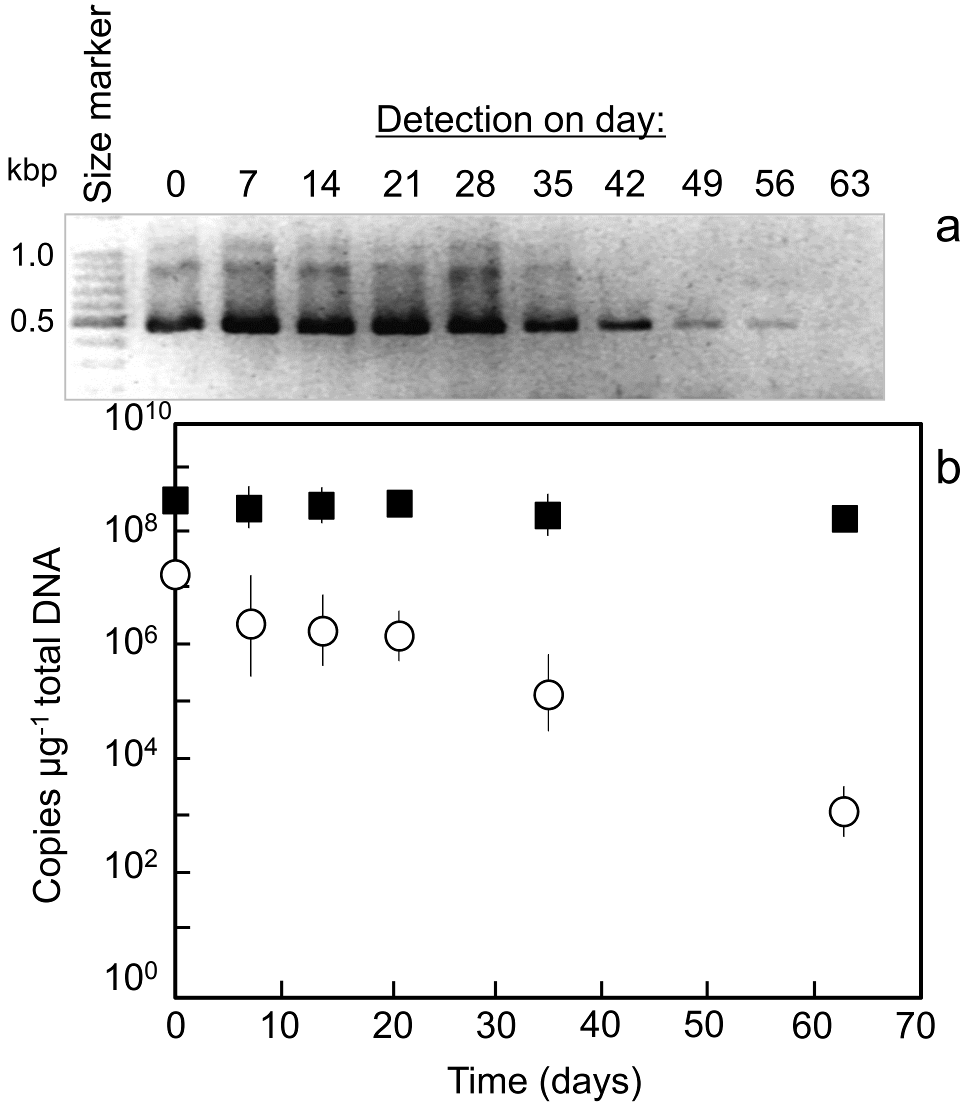

Size marker
Detection on day:
kbp
0 7 14 21 28 35 42 49 56 63
a
1.0
0.5
b
10^{10}
10^{8}
10^{6}
10^{4}
10^{2}
10^{0}
Copies μg^{-1} total DNA
0 10 20 30 40 50 60 70
Time (days)

Fig. S3

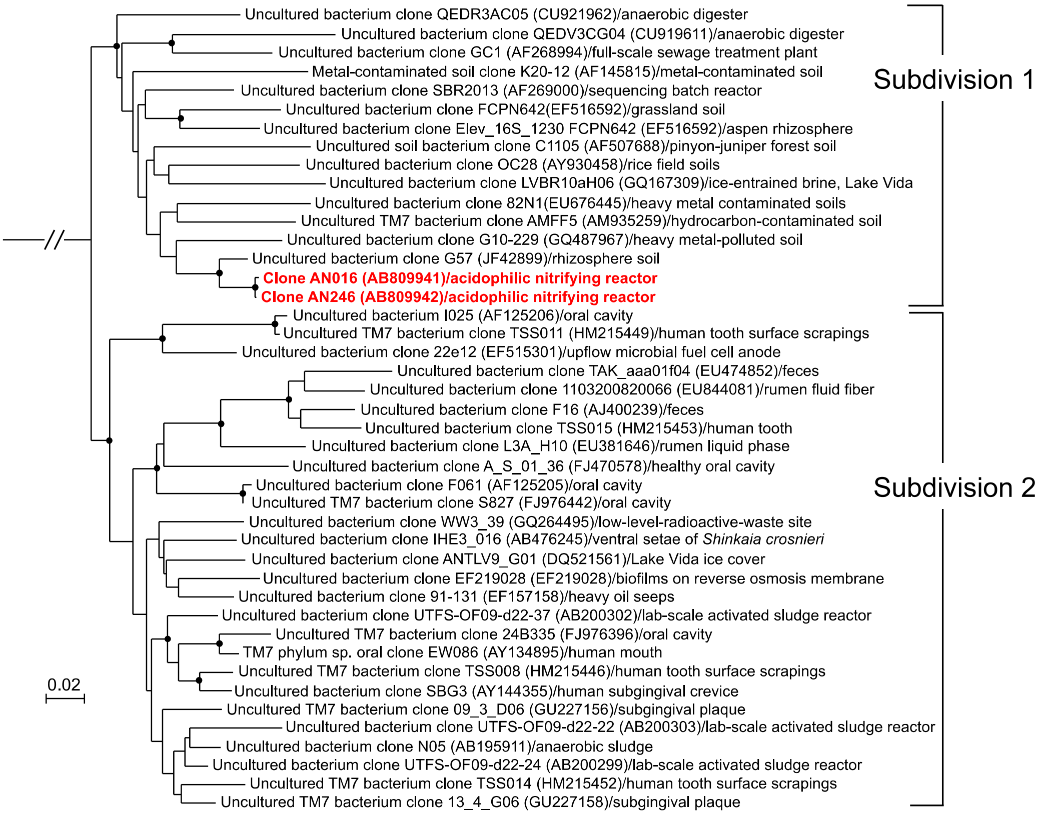
Uncultured bacterium clone QEDR3AC05 (CU921962)/anaerobic digester
Uncultured bacterium clone QEDV3CG04 (CU919611)/anaerobic digester
Uncultured bacterium clone GC1 (AF268994)/full-scale sewage treatment plant
Metal-contaminated soil clone K20-12 (AF145815)/metal-contaminated soil
Uncultured bacterium clone SBR2013 (AF269000)/sequencing batch reactor
Uncultured bacterium clone FCPN642(EF516592)/grassland soil
Uncultured bacterium clone Elev_16S_1230 FCPN642 (EF516592)/aspen rhizosphere
Uncultured soil bacterium clone C1105 (AF507688)/pinyon-juniper forest soil
Uncultured bacterium clone OC28 (AY930458)/rice field soils
Uncultured bacterium clone LVBR10aH06 (GQ167309)/ice-entrained brine, Lake Vida
Uncultured bacterium clone 82N1(EU676445)/heavy metal contaminated soils
Uncultured TM7 bacterium clone AMFF5 (AM935259)/hydrocarbon-contaminated soil
Uncultured bacterium clone G10-229 (GQ487967)/heavy metal-polluted soil
Uncultured bacterium clone G57 (JF42899)/rhizosphere soil
Clone AN016 (AB809941)/acidophilic nitrifying reactor
Clone AN246 (AB809942)/acidophilic nitrifying reactor
Uncultured bacterium I025 (AF125206)/oral cavity
Uncultured TM7 bacterium clone TSS011 (HM215449)/human tooth surface scrapings
Uncultured bacterium clone 22e12 (EF515301)/upflow microbial fuel cell anode
Uncultured bacterium clone TAK_aaa01f04 (EU474852)/feces
Uncultured bacterium clone 1103200820066 (EU844081)/rumen fluid fiber
Uncultured bacterium clone F16 (AJ400239)/feces
Uncultured bacterium clone TSS015 (HM215453)/human tooth
Uncultured bacterium clone L3A_H10 (EU381646)/rumen liquid phase
Uncultured bacterium clone A_S_01_36 (FJ470578)/healthy oral cavity
Uncultured bacterium clone F061 (AF125205)/oral cavity
Uncultured TM7 bacterium clone S827 (FJ976442)/oral cavity
Uncultured bacterium clone WW3_39 (GQ264495)/low-level-radioactive-waste site
Uncultured bacterium clone IHE3_016 (AB476245)/ventral setae of *Shinkaia crosnieri*
Uncultured bacterium clone ANTLV9_G01 (DQ521561)/Lake Vida ice cover
Uncultured bacterium clone EF219028 (EF219028)/biofilms on reverse osmosis membrane
Uncultured bacterium clone 91-131 (EF157158)/heavy oil seeps
Uncultured bacterium clone UTFS-OF09-d22-37 (AB200302)/lab-scale activated sludge reactor
Uncultured TM7 bacterium clone 24B335 (FJ976396)/oral cavity
TM7 phylum sp. oral clone EW086 (AY134895)/human mouth
Uncultured TM7 bacterium clone TSS008 (HM215446)/human tooth surface scrapings
Uncultured bacterium clone SBG3 (AY144355)/human subgingival crevice
Uncultured TM7 bacterium clone 09_3_D06 (GU227156)/subgingival plaque
Uncultured bacterium clone UTFS-OF09-d22-22 (AB200303)/lab-scale activated sludge reactor
Uncultured bacterium clone N05 (AB195911)/anaerobic sludge
Uncultured bacterium clone UTFS-OF09-d22-24 (AB200299)/lab-scale activated sludge reactor
Uncultured TM7 bacterium clone TSS014 (HM215452)/human tooth surface scrapings
Uncultured TM7 bacterium clone 13_4_G06 (GU227158)/subgingival plaque
Subdivision 1
Subdivision 2
0.02

Supplemental table

Table S1. Phylogenetic assignment of the PCR-DGGE clones from ANSBR 1 by homology search with the BLAST and RDP-11 Seqmatch algorithms.

DGGE clone	Phylogenetic group assinged	BLAST sequence match		RDP sequence match	
		Closest relative (accession number)	% similarity	Closest relative as the type strain (accession number)	% similarity
a	*Chloroflexi*	Uncultured bacterium Amb_16S_837 (EF018570)	89	*Caldilinea aerophila* STL-6-01^T (AB067647)	81
b	*Gammaproteobacteria*	Uncultured bacterium FCPT619 (EF515934)	100	*Rhodanobacter ginsengisoli* GR17-7^T (EF166075)	92
c	*Gammaproteobacteria*	*Dokdonella ginsengisoli* Gsoil 191^T (AB245362)	95	*Dokdonella ginsengisoli* Gsoil 191^T (AB245362)	92
d	TM7	Uncultured candidate division TM bacterium Skagen89 (DQ64071)	96		
e	*Gammaproteobacteria*	Uncultured bacterium 8 (DQ011842)	100	*Rhodanobacter ginsengisoli* GR17-7^T (EF166075)	91
f	*Actinobacteria*	*Rhodococcus wratislaviensis* NCIMB 13082^T (Z37138)	100	*Rhodococcus wratislaviensis* NCIMB 13082^T (Z37138)	100
g	*Alphaproteobacteria*	*Acidicella facilis* ATCC 35904^T (D30774)	98	*Acidocella facilis* ATCC 35904^T (D30774)	98
